# Challenges in Diagnosing Biopsy-Negative Cutaneous Leishmaniasis and Role of Systemic Therapies

**DOI:** 10.7759/cureus.84396

**Published:** 2025-05-19

**Authors:** Soomal Rafique, Arisha Rafique, Adil Rahu, Sara Shafi, Alvera Rajper

**Affiliations:** 1 Internal Medicine, Southern Illinois University School of Medicine, Springfield, USA; 2 Internal Medicine, Liaquat University of Medical and Health Sciences, Hyderabad, PAK; 3 Internal Medicine, Dow University of Health Sciences, Civil Hospital Karachi, Karachi, PAK; 4 Internal Medicine, Jinnah Medical and Dental College, Karachi, PAK; 5 Internal Medicine, Sindh Institute of Urology and Transplantation, Karachi, PAK

**Keywords:** cutaneous leishmaniasis, endemic, histopathology, leishmaniasis, vector-borne diseases

## Abstract

Cutaneous leishmaniasis (CL) typically manifests as one or more chronic, non-healing skin ulcers, often with raised borders and central necrosis, accompanied by erythema. These lesions are most commonly seen on exposed areas. However, the clinical presentation of CL can vary based on factors such as the *Leishmania* species, the host’s immune response, and the region of infection. Biopsy typically confirms the diagnosis of CL by identifying amastigotes, the intracellular form of the parasite, in tissue samples. However, there are rare instances where a biopsy fails to detect the amastigotes, resulting in a negative biopsy despite a clinical presentation highly suggestive of CL. This scenario complicates the diagnostic process and may lead to delays in proper treatment.

We present the case of a middle-aged gentleman from a desert region of the country who had several weeks of hand lesions unresponsive to antibiotic therapy. Although a biopsy of the lesions was negative, he was clinically diagnosed with CL based on positive *Leishmania* serology and the gross appearance of the lesions. There was no evidence of systemic involvement on bone marrow biopsy, and the lesions significantly improved with treatment using amphotericin B and miltefosine.

## Introduction

Cutaneous leishmaniasis (CL) is a parasitic infection caused by *Leishmania* species, which is transmitted through the bite of infected sandflies [1]. It is characterized by ulcerative skin lesions, often occurring in regions endemic to leishmaniasis [2]. Although the diagnosis of CL is typically confirmed through skin biopsy [3], cases where biopsy results are negative pose a diagnostic challenge. Our case explores the complexities of biopsy-negative CL, the differential diagnosis, and the importance of a multidisciplinary approach to diagnosis and treatment. Biopsy-negative CL remains a diagnostic challenge, but clinical suspicion should persist in endemic areas or with typical features.

## Case presentation

A 41-year-old man with no medical history presented to the clinic with complaints of a rash that had developed on his hands over the past month. The rash began as round, itchy lesions, which gradually progressed to reveal rough, scaly patches. Notably, the lesions were confined to his hands, with no other lesions elsewhere on the body and no associated systemic symptoms. The patient worked as a farmer in Balochistan, the desert region of Pakistan. Medical attention was sought when the lesions became painful. There was no history of fevers, chills, weight loss, or other systemic signs. The patient could not recall any recent insect bite.

**Figure 1:**
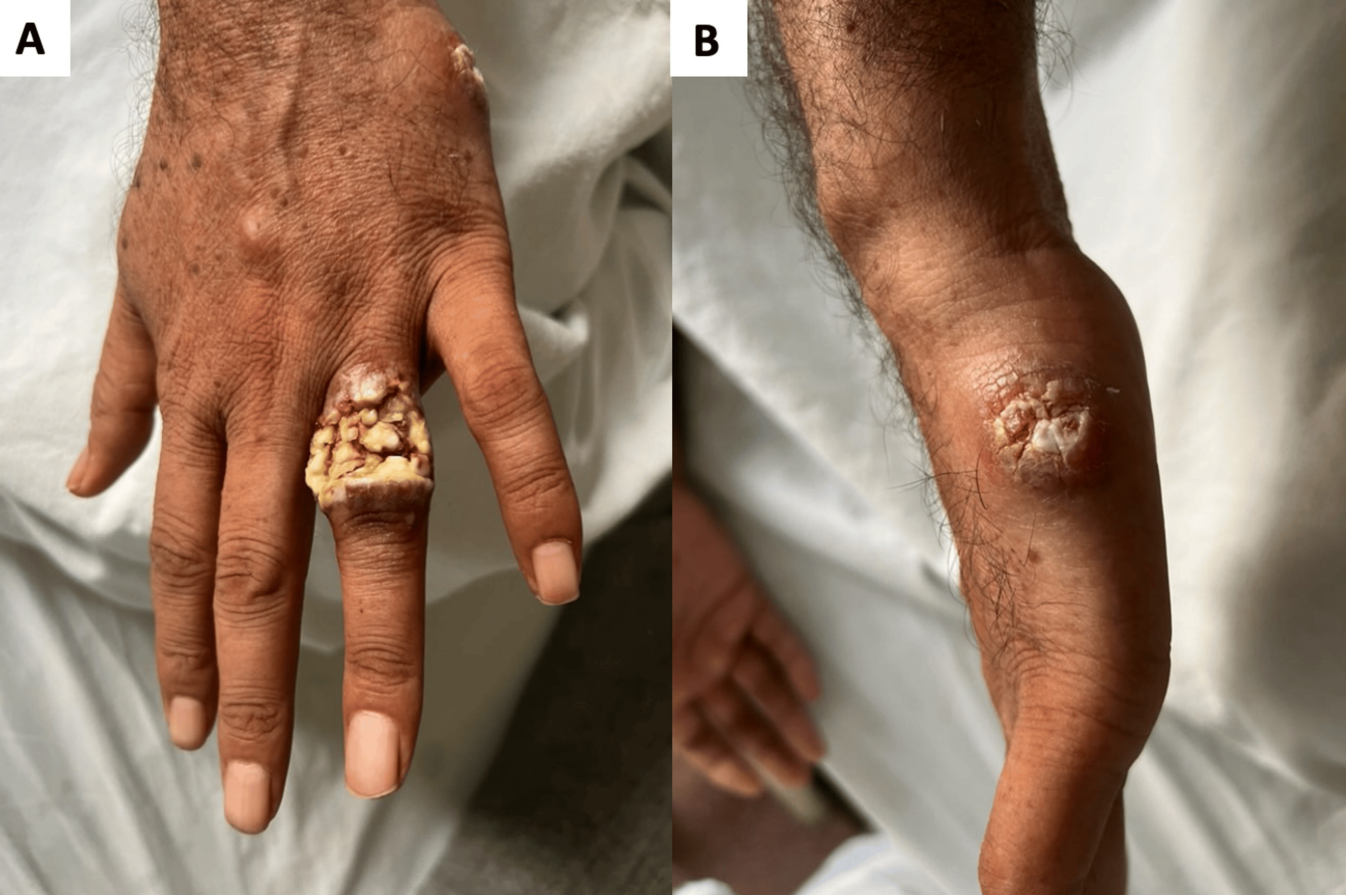
Ulcerated nodular lesions, with a scaly, verrucous surface, observed on the third finger (A) and along the ulnar border of the left hand (B).

Upon physical examination, two lesions were noted on the left hand, measuring 2 × 1 cm on the ring finger (Figure 1A) and another circumferential 1 cm lesion on the lateral border (Figure 1B). The lesions were round with a scaly, verrucous surface and an erythematous base. The lesion on the ring finger was more ulcerated, and the one on the lateral border was more nodular with underlying hard swelling.

The patient’s vital signs were within normal limits. The lesions were initially treated as skin abscesses and infected arthropod bites with multiple antibiotics. With no improvements in the lesions, several differentials were sought, including CL, fungal infections, cutaneous tuberculosis, and sarcoidosis. A skin biopsy was planned; however, given the characteristic appearance of CL, serologic tests were also ordered. Work-up revealed positive *Leishmania donovani* IgG antibodies. Pathology testing was performed on the skin scrapings, which did not reveal *Leishmania donovani* bodies. The patient underwent a bone marrow biopsy, which returned negative for visceral leishmaniasis. He was started on intravenous amphotericin B 70 mg once daily (1 mg/kg/day) during hospitalization for 15 days, along with oral miltefosine 50 mg three times daily for four weeks. At a follow-up visit one month later, the lesions showed complete recovery following the completion of therapy.

## Discussion

The worldwide incidence of CL is significant, with an estimated 0.7 to 1.2 million new cases occurring annually [4]. The Centers for Disease Control and Prevention reports that CL is endemic in 87 countries across six continents, with an estimated annual prevalence of 4.13 million cases, including 700,000 new cases globally [4]. CL is endemic in diverse regions such as the Americas, the Mediterranean basin, and western Asia, from the Middle East to Central Asia [5,6]. The disease is highly prevalent in Pakistan, particularly in the northern and western regions, which is where our patient originated [7]. The number of CL cases diagnosed in Balochistan, Pakistan, from August 2018 to December 2019 was 4,072. This data was derived from a study that analyzed clinically suspected cases of CL in the region [8].

The diagnosis of CL involves a combination of clinical evaluation and laboratory testing. According to the Infectious Diseases Society of America (IDSA) and the American Society of Tropical Medicine and Hygiene (ASTMH), CL should be considered in patients with compatible skin lesions and a history of exposure in a leishmaniasis-endemic area [9]. Clinically, CL presents as chronic skin lesions that are usually painless, with well-defined and often indurated borders. These lesions can be nodular or ulcerative and typically occur on exposed areas of the skin [9], as seen in our patient. Laboratory evaluation includes tissue smears, culture, polymerase chain reaction (PCR), and rapid diagnostic tests [9]. The choice of diagnostic method depends on lesion characteristics, available laboratory support, and the suspected *Leishmania* species [9].

Diagnostic challenges arise when biopsy results are negative due to low parasite load, histopathological variability, or suboptimal sampling techniques [9]. Proper specimen handling and preparation are crucial for accurate diagnosis [9,10], and failure in this regard was likely the reason for negative findings in our case. When traditional methods such as smear, culture, or histopathology are inconclusive, molecular techniques such as PCR and immunohistochemistry can significantly improve diagnostic yield. PCR is particularly sensitive and can detect *Leishmania* DNA even in cases with low parasite burden [9,11]. The IDSA and ASTMH recommend using a combination of diagnostic methods, including PCR and immunohistochemistry, to improve accuracy in challenging cases [11]. Given the improvement in skin lesions with amphotericin B and miltefosine, further testing was deferred in our case. As demonstrated in our patient, where the biopsy was negative but clinical suspicion remained high, a strong index of suspicion based on clinical presentation and travel history is essential for initiating empiric treatment in endemic areas or cases with a high pretest probability of CL [3].

Once CL is confirmed or strongly suspected, treatment strategies are based on lesion severity, *Leishmania* species, and the patient’s immune status [3]. In biopsy-negative cases, clinicians must rely on clinical judgment, epidemiological context, and diagnostic support tools to guide management. Treatment options include systemic therapies such as pentavalent antimonials (e.g., sodium stibogluconate), liposomal amphotericin B, and miltefosine. Localized lesions may be treated with intralesional antimonials or cryotherapy [3]. Local therapy is usually preferred for uncomplicated CL and includes intralesional pentavalent antimonials. This approach reduces systemic side effects and is more cost-effective, although it requires expertise in administration [12].

The failure rate of intralesional antimonials in treating localized CL varies significantly by geography and *Leishmania* species, for example, 17.2% in Brazil [13] compared to 40.3% in Ethiopia [14]. According to a systematic review by Brito et al., the pooled efficacy rate of intralesional pentavalent antimonials is approximately 75% (95% CI = 68-81%), implying a failure rate of about 25% [12]. These variations underscore the importance of considering geographic and species-specific factors when evaluating treatment outcomes. For refractory or complex cases, liposomal amphotericin B has proven effective and offers a shorter duration of intravenous administration along with a better safety profile compared to sodium stibogluconate [15]. Miltefosine is also a Food and Drug Administration-approved treatment for CL caused by *Leishmania (V.) braziliensis*, *Leishmania (V.) panamensis*, and *Leishmania (V.) guyanensis* [3]. Due to the unavailability of intralesional pentavalent antimonials, systemic therapy was chosen for our patient, resulting in complete recovery.

## Conclusions

Biopsy-negative CL presents a diagnostic challenge but should not preclude the consideration of this condition in endemic areas or in patients with typical clinical features. A negative biopsy does not rule out the diagnosis, and clinicians should adopt a comprehensive approach that includes clinical examination, epidemiological context, molecular diagnostics, and serological testing. In conclusion, the case of biopsy-negative CL highlights the need for heightened clinical awareness and a multidisciplinary approach to ensure timely and accurate diagnosis. With a comprehensive diagnostic workup, even in the absence of a definitive biopsy, it is possible to initiate effective treatment and prevent further complications associated with this disease.
